# Exercise motives among university students – A Brazil-Portugal transnational study

**DOI:** 10.3389/fpsyg.2022.1009762

**Published:** 2022-11-14

**Authors:** Lucas Arrais Campos, Adrielly dos Santos, Mafalda Margarida Basto Sampaio, João Marôco, Juliana Alvares Duarte Bonini Campos

**Affiliations:** ^1^Faculty of Medicine and Health Technology, Tampere University, Tampere, Finland; ^2^Tampere University Hospital, Tampere, Finland; ^3^Department of Morphology and Children’s Clinic, School of Dentistry, São Paulo State University (UNESP), Araraquara, Brazil; ^4^Department of Biological Sciences, School of Pharmaceutical Sciences, São Paulo State University (UNESP), Araraquara, Brazil; ^5^William James Center for Research (WJCR), University Institute of Psychological, Social, and Life Sciences (ISPA), Lisbon, Portugal

**Keywords:** motivation, exercise, psychometrics, validation study, university students

## Abstract

**Background:**

Identifying the motives why people exercise is interesting for the planning of effective health promoting strategies.

**Objectives:**

To estimate the psychometric properties of the exercise motivations inventory (EMI-2) in Brazilian and Portuguese university students, and to compare motive-related factors for exercise among students.

**Methods:**

One thousand Brazilian (randomly splitted into “Test sample” [*n* = 498] and “Validation sample” [*n* = 502]) and 319 Portuguese students participated in this cross-sectional study. Motives for exercise were evaluated using EMI-2. Exploratory factor analysis was performed in the test sample. Then, confirmatory factor analysis was performed in the validation and Portuguese samples. The EMI-2 scores were compared according to sex, exercise, and weight status (ANOVA, *α* = 5%).

**Results:**

EMI-2 factor model was explained by 5 factors and presented adequate fit (*χ*^2^/*df* ≤ 3.2; CFI ≥ 0.9; TLI ≥ 0.9; RMSEA ≤ 0.07; and *α* ≥ 0.83). The motives for exercising were mainly related to psychological and interpersonal factors for men, health-related factors for women, and body-related factors for overweight and obese individuals. People who practice exercise had higher EMI-2 scores.

**Conclusion:**

The 5-factor model is suggested for a comprehensive assessment of motives for exercise. Individual characteristics should be considered for development of tailored protocols.

## Introduction

Exercise has been related to well-being, lower risk of chronic conditions related to sedentarism, and satisfaction with body image (Gill et al., 2017; Hu et al., 2020; Pedersen et al., 2021; Katzmarzyk et al., 2022). Engaging in exercise depends on individual motivation (Morris and Roychowdhury, 2020), which, according to Deci and Ryan’s self-determination theory (Ryan and Deci, 2000), is defined as a factor that determines a specific behavior. A suggested application for self-determination theory is the identification of motives for exercise, which are considered as reflecting extrinsic and intrinsic motivations (Valenzuela et al., 2020). Extrinsic motives are related to doing exercise for instrumental reasons or to obtain results that are external to the exercise *per se*, such as obtaining a reward or avoiding disapproval from others (Markland and Ingledew, 1997). Intrinsic motives are related to the enjoyment or the challenge of practicing an exercise (Markland and Ingledew, 1997).

The identification of the motives for practicing exercise is relevant for the planning of effective health promoting strategies in target populations (Valenzuela et al., 2020; Pedersen et al., 2021). It can be an important information for health professionals and institutional managers to develop and maintain a healthy lifestyle in target populations, encompassing the exercise adherence of individuals. Because of the abstract nature of the concept, the motives for exercise are measured and evaluated by specific measuring instruments (psychometric scales), such as the exercise motivations inventory (EMI; Markland and Hardy, 1993; Markland and Ingledew, 1997).

The EMI was originally developed for English-speaking exercisers (Markland and Hardy, 1993) and then underwent some adaptations for broadening the assessment of motive-related factors and extending its use to non-exercisers. This new version was named EMI-2 (Markland and Ingledew, 1997) and has been translated, adapted, and used in several countries and populations (Ingledew and Sullivan, 2002; Cho et al., 2020; Kim and Cho, 2022; Vuckovic et al., 2022) including Portugal (Alves and Lourenço, 2003) and Brazil (Klain et al., 2015). As any psychometric instrument, before applying the EMI-2 to a specific population, its psychometric properties should be assessed in a sample of the population (Marôco, 2021a) to ensure that the collected data is valid and reliable.

Previous studies assessed the psychometric properties of the EMI-2 (Markland and Ingledew, 1997; Ingledew and Sullivan, 2002; Klain et al., 2015). However, as can be seen in the study by Klain et al. (2015), the analyses to verify these properties were performed considering each factor of the EMI-2 separately, without assessing the correlation between motive-related factors for exercising. This does not reflect the original proposal of the concepts measured by the EMI-2 (Markland and Ingledew, 1997), which points to a conceptual relationship existing between the EMI-2 motive-related factors and even the possibility of clustering (second-order hierarchical factor). For this reason, Rodrigues et al. (2019) evaluated the EMI-2 psychometric properties considering it a single factor model (first-order oblique model and second-order hierarchical model) in a sample of Portuguese exercisers. Nevertheless, there is still a gap in the literature on the evaluation of the psychometric properties of EMI-2 factorial model that considers the motive-related factors simultaneously in a sample of non-exercisers. Therefore, studies aiming at this are relevant to verify the applicability of the EMI-2, as well as the validity and reliability of the results, in different target populations.

In addition, the academic community, including university students, has an important social responsibility since it contributes for the establishment and dissemination of values in a society (Smolentseva, 2022). Thus, the investigation of the motive-related factors for the practice of exercise in this population could be helpful not only to develop specific strategies for encouraging exercise among students, but also to understand how motive-related factors of students reflect in the general population. Additionally, exploring different cultures will generate comprehensive and relevant evidence and expand discussions internationally, which may contribute to the development of strategies to promote a healthy life style (Pedersen et al., 2021).

Studies indicate that characteristics such as sex, weight status, and level of exercise may also influence the motives for exercise (Ingledew and Sullivan, 2002; Lores et al., 2004; Guedes et al., 2013; Ednie and Stibor, 2017; Ley, 2020; Anic et al., 2022; Vuckovic et al., 2022). Among young men and women, the main motive for exercising is related to physical appearance (Ednie and Stibor, 2017; Anic et al., 2022), competitiveness (Guedes et al., 2013), and sociability (Ley, 2020; Valenzuela et al., 2020). Body mass index is positively related to weight management motives (Ingledew and Sullivan, 2002; Anic et al., 2022) and negatively related to the other factors of EMI-2 (Ingledew and Sullivan, 2002). For athletes and people in advanced-level sports, motives are linked to competition and socialization (Lores et al., 2004; Vuckovic et al., 2022), while among beginners, motives are related to appearance and body image (Lores et al., 2004).

This transnational study was carried out to confirm a factorial model for EMI-2 when applied to university students considering the correlation between the scale’s 14 factors and preserving the theoretical framework of the instrument. Thus, the study objectives were (i) to estimate the psychometric properties of the EMI-2 in a sample of Brazilian and Portuguese university students; (ii) to identify a factorial model that considers all the EMI-2 concepts simultaneously and evaluate its psychometric properties; and (iii) to identify and compare the motive-related factors for exercise according to sex, exercise level, and weight status among students considering each country separately.

## Materials and methods

### Study design and sampling

This was an observational cross-sectional study with non-probability sampling conducted to carry out a transnational Brazil-Portugal comparison. Students enrolled at one Brazilian university and at five Portuguese institutions were invited to participate in the study. The selected university in Brazil offers courses from different major areas of knowledge (Humanities and Social sciences, Biology and Health sciences, and Exact sciences) and has a high number of enrolled students, the reason why only one institution was included. As in Portugal institutions have a reduced number of courses, generally from the same area of knowledge, data collection was done in more institutions.

Initially, the minimum sample size was calculated following the proposal of Hill and Hill (2008), who recommend a minimum of 5 individuals per item of the instrument. EMI-2 has 51 items, therefore, the minimum sample size required in each country was 255 individuals. After the establishment of a new theoretical model for the sample, a new minimum sample size was calculated to confirm if the sample size initially estimated would adequately contemplate what is required in this new model. For sample size calculation, the parameters to be estimated in the factorial model were considered and the Monte-Carlo simulation was performed as described by Brown (2015) using the criteria defined by Muthén and Muthén (2017): (i) bias of parameter estimates smaller than 10%; (ii) coverage of 95% confidence intervals larger than 91% and (iii) percentage of significant coefficients (power) larger or equal to 80%. Mplus software (version 8. Muthén & Muthén, Los Angeles) was used for 1,000 simulations for sample sizes of 100, 200 and 300, defining as 200 the adequate sample size.

### Study variables

Information about sex, age (years), use of weight control medication (no, yes) and supplements (no, yes), body mass index (BMI), and exercise level was collected. BMI was calculated from the self-reported weight (kg) and height (m) and used for weight status classification (Piqueras et al., 2021). The typical exercise practice was assessed through the questions “Do you exercise (no/yes)” and “How often do you exercise (days/week).”

### Measuring scale

Motives for exercise were assessed through the EMI-2. The tool was originally proposed in English by Markland and Ingledew (1997) and consists of 51 items divided into 14 motive-related factors (“Stress Management”: items 6, 20, 34, and 46, “Revitalization”: items 3, 17 and 31, “Enjoyment”: items 9, 23, 37 and 48, “Challenge”: items 14, 28, 42 and 51, “Social Recognition”: items 5, 19, 33 and 45, “Affiliation”: items 10, 24, 38 and 49, “Competition”: items 12, 26, 40 and 50, “Health Pressures”: items 11, 25 and 39, “Ill health avoidance”: items 2, 16 and 30, “Positive Health”: items 7, 21 and 35, “Weight Management”: items 1, 15, 29 and 43, “Appearance”: items 4, 18, 32 and 44, “Strength and Endurance”: items 8, 22, 36 and 47 and “Nimbleness”: items 13, 27 and 41) grouped into 5 domains (“Psychological,” “Interpersonal,” “Health,” “Body,” and “Physical Condition”). The EMI-2 responses are scored in a 6-point Likert-type scale and range from 0 (“Not at all true to me”) to 5 (“Very true to me”).

EMI-2 was translated and adapted to the Portuguese language by Alves and Lourenço (2003), considering context of Portugal. Klain et al. (2015), through face and content validity and expert panel, made some changes in this Portuguese version (Alves and Lourenço, 2003) aiming to adapt the EMI-2 for Brazilian context. In the present study, initially both EMI-2 Portuguese versions were evaluated and compared by the researchers (Brazilian: AS and JADBC; Portuguese: MMB and JM). It was observed that the adaptation made by Klain et al. (2015) dealt with minor semantic and spelling changes obtaining an adapted version that meets the orthographic agreement established between Portuguese-speaking countries in 2009 and preserves semantic and cultural equivalence in both Brazil and Portugal. Thus, in the present study we used the Portuguese version of the EMI-2 adapted by Klain et al. (2015) (Supplementary File 1) in both Brazil and Portugal, since it was considered adequate by the researchers for its application in the Lusophony context of both countries.

### Psychometric properties

The psychometric sensitivity of the EMI-2 was estimated from measures of central tendency, variability, and distribution of responses. Absolute values of skewness (Sk) ≤3 and kurtosis (Ku) ≤7 were indicative of non-severe violation of the normal distribution of the data, which confirms the psychometric sensitivity of the items (Marôco, 2021a).

Initially, a confirmatory factor analysis (CFA) was conducted considering each of the 14 factors individually (14 single-factor models) as proposed by Markland and Ingledew (1997) and Klain et al. (2015). The robust Weighted Least Squares Mean and Variance Adjusted estimation method was used. For the quality of fit, the Chi-square for degrees of freedom ratio (*χ*^2^/*df*), the comparative fit index (CFI), the Tucker-Lewis index (TLI), the standardized root mean squared residual (SRMR), and the root mean square error of approximation (RMSEA) were used. The factor loading (*λ*) of the items was also considered. The fit was considered adequate when *λ* ≥ 0.50, *χ*^2^/*df* ≤ 2.0, CFI and TLI ≥ 0.90, and SRMR and RMSEA ≤ 0.08 (Marôco, 2021a).

Then, the model fit to the sample with 14 correlated factors was tested. As the polychoric matrix did not converge, an exploratory strategy was used to verify whether the 5-factor model, as also originally proposed by Markland and Ingledew (1997), could be considered appropriate for the study sample. An exploratory factor analysis (EFA) was performed to estimate the factors based on the data and then a theoretical evaluation of the items’ content grouped by factor was performed to verify their adequacy. The Brazilian sample, which was large enough to allow further confirmation of the obtained proposal, was randomly splitted into two subsamples (test sample: *n* = 498, validation sample: *n* = 502).

For EFA, the principal component analysis followed by Varimax rotation was used. The adequacy of the sample to perform EFA was assessed using the KMO index, being considered adequate if > 0.7. Common factors with eigenvalues greater than 1 were retained and items with a factor loading ≥0.40 (Marôco, 2021b) were considered. Items that were not allocated within the original theoretical framework (1997) were removed.

After establishing the new factorial model, CFA was performed to confirm its adequacy in the Validation sample using the same quality assessment indices described above. For the refinement of the models we used the modification indices calculated by the Lagrange Multiplier (LM) method; LM values >11 were inspected (Marôco, 2021a). The new factor model was also fitted to the Portuguese sample and its adequacy assessed by CFA.

Convergent validity was estimated from the average variance extracted (AVE) as described by Fornell and Larcker (1981). Values of AVE ≥ 0.50 (Fornell and Larcker, 1981; Marôco, 2021a) indicated adequate convergent validity. Discriminant validity was assessed using correlation analysis between factors; discriminant validity was considered when AVE_i_ and AVE_j_ ≥ *r*_ij_^2^ (Fornell and Larcker, 1981; Marôco, 2021a).

Reliability was assessed by the composite reliability (CR) as previously proposed (Fornell and Larcker, 1981), and internal consistency (Marôco, 2021a) using the ordinal alpha coefficient (*α*). CR and *α* ≥ 0.70 were considered indicative of adequate reliability.

Psychometric properties analyses were performed using the “lavaan” (Rosseel, 2012) and “semTools” (Jorgensen et al., 2022) packages of the R program (R Core Team, 2022).

### Comparison of mean score of the EMI-2 factors

The mean score (arithmetic mean of the responses) for each factor (single-factor models and the 5-factor model) was calculated for each category of the variables of interest (sex, exercise practice, and weight status) according to the country. The assumptions of normality and homoscedasticity were evaluated. For variables with *n* ≥ 80, normality was assessed by the shape of the distribution. Absolute values of skewness and kurtosis below 3 and 10, respectively, were indicative of approximation to the normal distribution (Kline, 2016). For categories of variables with *n* < 80, normality was assessed using Kolmogorov–Smirnov test. If data did not show approximation to the normal distribution, the comparisons were performed using Kruskal–Wallis test followed by Dunn *post-hoc* test. If data showed approximation to the normal distribution, the homoscedasticity of the factor score in the different categories was evaluated using the Levene’s test. If data showed homoscedasticity, the ANOVA followed by Tukey *post-hoc* test was used. If heteroscedasticity was observed, Welch’s ANOVA followed by Games-Howell *post-hoc* test was used. For decision making, a significance level of 5% was adopted. Data analyses were performed using the IBM SPSS Statistics 22 software (IBM Corp., Armonk, N.Y., United States).

### Procedures and ethical aspects

All procedures performed in the study were in accordance with the ethical standards of the institutional and/or national research committee and with the 1964 Helsinki Declaration and its later amendments or comparable ethical standards. In Brazil, the study was approved by the Research Ethics Committee of the Faculty of Pharmaceutical Sciences - UNESP (CAAE: 63553516.4.0000.5426) and in Portugal, by the Ethics Committee of the ISPA Research Center – University Institute (D/009/10/2018).

Students who agreed to participate and signed the informed consent form were included in the study. The general characteristics questionnaire and the EMI-2 were self-administered in the classroom, after written permission of the professor for using 10 to 15 min of his class in a pre-set day and time. Data collection in both countries was carried out in 2018 over a period of 6 months in Brazil and in 30 days in Portugal. A study flowchart is presented in Supplementary File 2.

## Results

A total of 1,319 university students participated in the study (Brazilians: *n* = 1,000; Portuguese: *n* = 319). Among the Brazilian participants, 67.4% (*n* = 674) were female and the average age was 21.08 ± 3.01 years, 691 (69.1%) were enrolled in Humanities/Social, 221 (22.1%) in Biology/Health, and 88 (8.8%) in Exact sciences. One hundred and sixty-nine (16.9%) of these students reported having taken weight control medication and 252 (25.6%), weight control supplements. Of the Brazilian students, 490 (49.2%) practiced exercise 3.5 ± 1.5 days/week. Regarding weight status, 94 (9.6%) were underweight, 628 (63.8%) eutrophic, and 262 (26.6%) overweight/obese.

Among the Portuguese, 219 (68.7%) were female and the average age was 22.07 ± 3.00 years, 168 (55.4%) were enrolled in Biological/Health, 121 (39.9%) in Humanities/Social, and 14 (4.7%) in the Exact sciences. Of these students, 29 (9.1%) reported having taken weight control medication and 73 (23.1%), weight control supplements. Two hundred and twenty (69.4%) practiced exercise 3.3 ± 1.5 days/week. Regarding weight status, 27 (8.8%) were underweight, 227 (73.9%) eutrophic, and 53 (17.3%) overweight/obese.

Table 1 shows the distribution of responses given to EMI-2 items by Brazilian and Portuguese students. No item presented severe violation of the assumption of normality (Sk < |3|, Ku < |7|) indicating, therefore, adequate psychometric sensitivity for the samples. Table 2 presents the fitting of the EMI-2 factors for the Brazilian and Portuguese samples, considering each factor as a single model. Factors had appropriate fit to the data. Table 3 presents the results obtained in the EFA performed with the Brazilian Test sample (KMO = 0.942). Considering the eigenvalue rule and the original theoretical framework, the factorial model for the motivation of exercise was explained by 5 factors.

**Table 1:** Distribution of responses given to exercise motivations inventory 2 (EMI-2) items by Brazilian (*n* = 1,000) and Portuguese (*n* = 319) students.

	Brazil				Portugal			
Item	Mean	Standard deviation	Skewness	Kurtosis	Mean	Standard deviation	Skewness	Kurtosis
EMI1	3.07	1.74	−0.49	−1.02	3.13	1.54	−0.67	−0.48
EMI2	3.46	1.53	−0.83	−0.27	3.13	1.58	−0.65	−0.63
EMI3	3.81	1.45	−1.17	0.42	4.20	1.06	−1.78	3.75
EMI4	1.46	1.62	0.77	−0.63	1.29	1.45	0.84	−0.38
EMI5	0.67	1.20	1.90	2.83	0.94	1.20	1.12	0.25
EMI6	1.94	1.76	0.39	−1.19	2.26	1.68	−0.10	−1.38
EMI7	4.04	1.29	−1.46	1.61	4.34	0.90	−2.00	5.95
EMI8	3.28	1.62	−0.67	−0.66	3.89	1.19	−1.26	1.44
EMI9	2.88	1.82	−0.33	−1.26	3.51	1.50	−0.96	0.05
EMI10	1.55	1.68	0.69	−0.86	1.71	1.56	0.39	−1.12
EMI11	1.84	1.86	0.42	−1.35	1.26	1.60	0.86	−0.72
EMI12	1.17	1.63	1.16	−0.02	1.92	1.66	0.30	−1.21
EMI13	2.62	1.76	−0.17	−1.26	3.37	1.35	−0.93	0.24
EMI14	2.05	1.80	0.28	−1.31	3.05	1.58	−0.62	−0.65
EMI15	2.78	2.06	−0.27	−1.58	2.58	1.89	−0.12	−1.46
EMI16	3.87	1.44	−1.26	0.70	3.74	1.32	−1.14	0.83
EMI17	3.07	1.72	−0.46	−1.04	3.60	1.34	−1.04	0.55
EMI18	3.69	1.55	−1.08	0.08	3.71	1.33	−1.15	0.83
EMI19	0.53	1.11	2.41	5.48	0.86	1.24	1.47	1.38
EMI20	3.14	1.70	−0.57	−0.90	3.05	1.65	−0.62	−0.80
EMI21	3.93	1.35	−1.35	1.19	4.09	1.10	−1.61	2.92
EMI22	3.61	1.53	−0.99	−0.03	3.85	1.16	−1.19	1.48
EMI23	2.91	1.79	−0.34	−1.23	3.50	1.51	−1.01	0.06
EMI24	1.63	1.68	0.68	−0.81	1.98	1.58	0.23	−1.11
EMI25	2.32	2.01	0.10	−1.61	1.86	1.79	0.37	−1.33
EMI26	0.93	1.46	1.50	1.08	1.49	1.55	0.70	−0.66
EMI27	2.66	1.80	−0.19	−1.31	2.88	1.53	−0.57	−0.67
EMI28	1.71	1.78	0.58	−1.08	2.82	1.57	−0.49	−0.86
EMI29	3.17	1.87	−0.61	−1.10	3.27	1.62	−0.74	−0.57
EMI30	3.15	1.81	−0.59	−1.03	2.84	1.70	−0.43	−1.07
EMI31	2.82	1.82	−0.31	−1.26	3.20	1.53	−0.81	−0.29
EMI32	3.28	1.74	−0.67	−0.85	3.46	1.45	−0.87	−0.03
EMI33	0.76	1.24	1.62	1.80	1.30	1.44	0.74	−0.67
EMI34	3.14	1.68	−0.56	−0.86	3.50	1.45	−1.02	0.30
EMI35	3.81	1.44	−1.20	0.61	4.18	1.00	−1.73	4.02
EMI36	2.85	1.77	−0.35	−1.19	3.59	1.40	−1.17	0.78
EMI37	2.52	1.81	−0.06	−1.35	3.28	1.61	−0.78	−0.52
EMI38	1.99	1.80	0.37	−1.23	2.26	1.65	0.01	−1.32
EMI39	1.44	1.78	0.85	−0.76	1.69	1.79	0.59	−1.09
EMI40	0.93	1.48	1.53	1.19	1.70	1.61	0.48	−0.99
EMI41	2.59	1.85	−0.15	−1.38	2.92	1.57	−0.46	−0.84
EMI42	2.60	1.80	−0.13	−1.33	2.95	1.60	−0.52	−0.85
EMI43	3.03	1.89	−0.46	−1.28	3.11	1.65	−0.58	−0.85
EMI44	2.84	1.84	−0.30	−1.29	3.13	1.63	−0.60	−0.79
EMI45	0.69	1.28	1.97	3.01	1.06	1.37	1.19	0.45
EMI46	3.14	1.75	−0.57	−0.95	3.58	1.42	−1.11	0.56
EMI47	2.89	1.78	−0.38	−1.18	3.57	1.36	−1.02	0.41
EMI48	2.82	1.86	−0.29	−1.33	3.50	1.57	−1.00	−0.04
EMI49	1.23	1.51	1.01	−0.13	1.29	1.45	0.85	−0.48
EMI50	1.08	1.50	1.28	0.52	1.88	1.68	0.36	−1.16
EMI51	1.81	1.79	0.50	−1.15	2.32	1.68	−0.01	−1.23

**Table 2:** Fit of the 14 factors of the exercise motivations inventory (EMI-2) as single-factor models to the samples from Brazil and Portugal.

CFA*						
Factor	*χ*^2^/*df*	CFI	TLI	RMSEA	SRMR	*λ*
Brazil (*n* = 1,000)						
Stress management	0.556	1.000	1.000	0.000	0.005	0.532–0.955
Revitalization	0.481	1.000	1.000	0.000	0.000	0.780–0.933
Enjoyment	0.598	1.000	1.000	0.000	0.003	0.843–0.922
Challenge	0.643	1.000	1.000	0.000	0.016	0.717–0.854
Social recognition	0.596	1.000	1.000	0.000	0.014	0.793–0.910
Affiliation	0.741	1.000	1.000	0.000	0.018	0.770–0.917
Competition	0.611	1.000	1.000	0.000	0.005	0.821–0.935
Health pressures	0.460	1.000	1.000	0.000	0.000	0.677–0.736
Ill health avoidance	0.482	1.000	1.000	0.000	0.000	0.781–0.937
Positive health	0.515	1.000	1.000	0.000	0.000	0.897–0.931
Weight management	0.685	1.000	1.000	0.000	0.011	0.751–0.939
Appearance	0.534	1.000	1.000	0.000	0.005	0.504–0.939
Strength and endurance	0.177	1.000	1.000	0.000	0.011	0.761–0.927
Nimbleness	0.514	1.000	1.000	0.000	0.000	0.671–0.966
Portugal (*n* = 319)						
Stress management	0.502	1.000	1.000	0.000	0.013	0.521–0.964
Revitalization	0.426	1.000	1.000	0.000	0.000	0.771–0.801
Enjoyment	0.607	1.000	1.000	0.000	0.012	0.821–0.902
Challenge	0.599	1.000	1.000	0.000	0.000	0.659–0.809
Social recognition	0.484	1.000	1.000	0.000	0.000	0.765–0.806
Affiliation	0.593	1.000	1.000	0.000	0.020	0.794–0.826
Competition	0.592	1.000	1.000	0.000	0.018	0.655–0.938
Health pressures	0.401	1.000	1.000	0.000	0.000	0.419–0.832
Ill health avoidance	0.435	1.000	1.000	0.000	0.000	0.735–0.856
Positive health	0.391	1.000	1.000	0.000	0.000	0.825–0.899
Weight management	0.581	1.000	1.000	0.000	0.014	0.683–0.906
Appearance	0.704	1.000	1.000	0.000	0.057	0.358–1.000
Strength and endurance	0.585	1.000	1.000	0.000	0.023	0.730–0.899
Nimbleness	0.447	1.000	1.000	0.000	0.000	0.561–0.974

*CFA, confirmatory factor analysis; λ, factor loading; *χ*^2^/*df*, Chi-square for degrees of freedom ratio; CFI, comparative fit index; TLI, Tuker-Lewis index; RMSEA, root mean square error of approximation; SRMR, standardized root mean squared residual.

**Table 3:** Factor loading of items and factors extracted in the exploratory factor analysis of the exercise motivations inventory 2 (EMI-2) using the Brazilian test subsample (*n* = 498).

	Factor				
Item	Psychological	Interpersonal	Health	Body	Physical condition
EMI3	0.682				
EMI6	0.437				
EMI7	0.447				0.429
EMI9	0.773				
EMI17	0.767				
EMI20	0.724				
EMI21	0.509		0.525		
EMI23	0.802				
EMI31	0.667				
EMI34	0.646				
EMI35	0.530				0.429
EMI37	0.707				
EMI46	0.719				
EMI48	0.704				
EMI5		0.585			
EMI10		0.469			
EMI12		0.760			
EMI14		0.489			
EMI19		0.718			
EMI24	0.437	0.508			
EMI26		0.802			
EMI28		0.669			
EMI33		0.702			
EMI38	0.494	0.513			
EMI40		0.765			
EMI45		0.742			
EMI49		0.459			
EMI50		0.755			
EMI51		0.504			
EMI2			0.530		
EMI11			0.652		
EMI16			0.664		
EMI25			0.748		
EMI30			0.731		
EMI39			0.595		
EMI1				0.793	
EMI15				0.777	
EMI18				0.640	0.430
EMI29				0.808	
EMI32				0.701	
EMI43				0.814	
EMI44				0.735	
EMI8					0.715
EMI13					0.629
EMI22	0.494				0.564
EMI27					0.645
EMI36					0.746
EMI41					0.688
EMI42		0.400			0.518
EMI47					0.582

For the proposed model considering the original theoretical framework, the items that were not allocated to the factor originally proposed were eliminated (e.g., if an item was allocated to measure the psychological factor, but our factor analysis allocated it to the interpersonal factor, the item was eliminated). Thus, 11 items (items 4, 7, 14, 21, 22, 24, 28, 35, 38, 42, 51) were deleted. The remaining items were distributed in the five domains (Psychological, Interpersonal, Health, Body, and Physical Condition), adequately contemplating the theoretical proposal.

**Figure 1:**
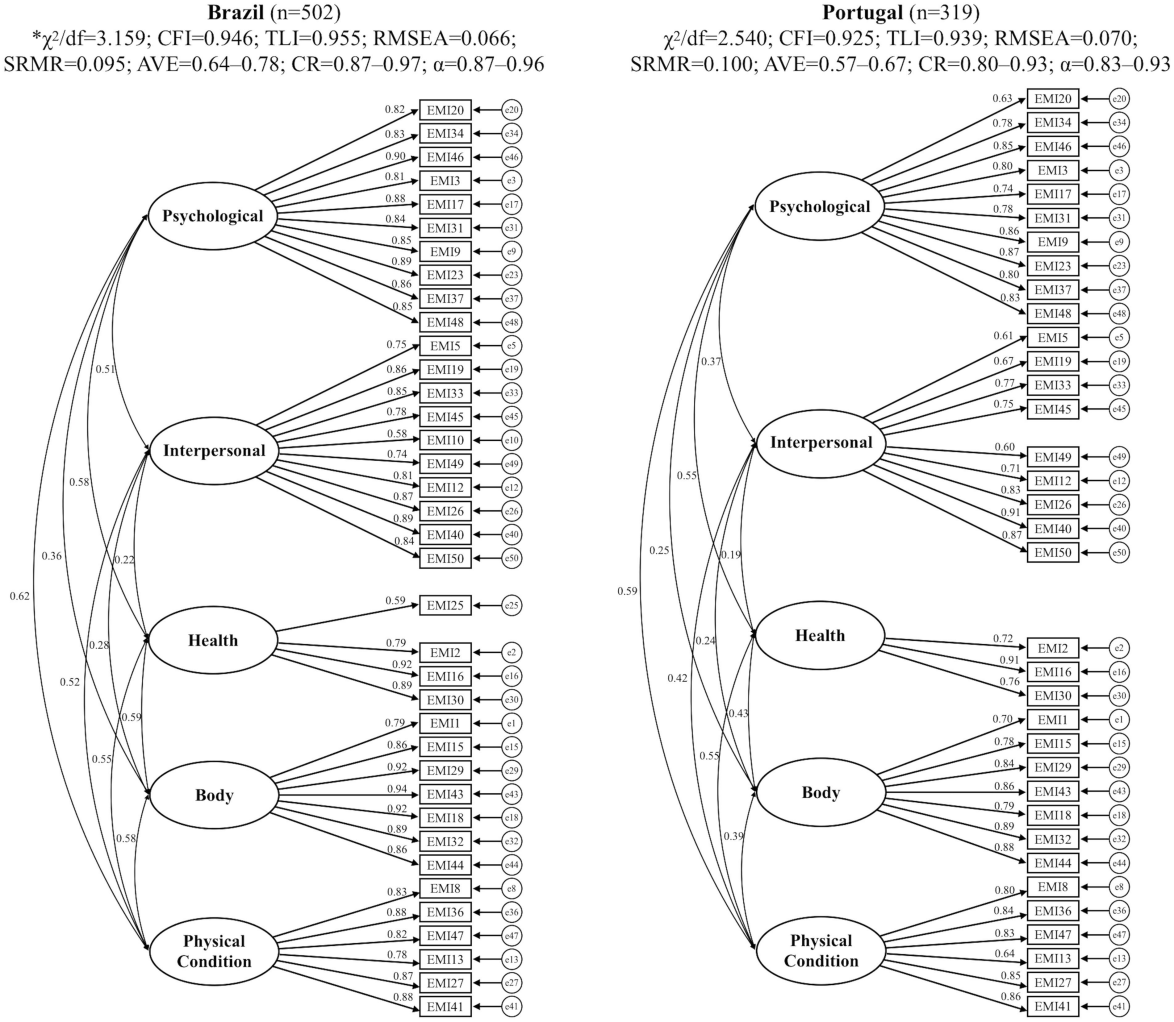
Factorial models fitted for the Brazilian and Portuguese samples.

Since no item displayed absolute skewness and kurtosis larger than |3| and |7| and the linearity was attested by residual analysis (residual plot) there was no violation of the assumptions that would limit the use of CFA. With the CFA of the new model proposed to the Brazilian sample (validation sample: *λ* = 0.368–0.936; *χ*^2^/*df* = 3.16; CFI = 0.938; TLI = 0.948; RMSEA = 0.066; SRMR = 0.099) and Portuguese (*λ* = 0.468–1,000; *χ*^2^/*df* = 3.65; CFI = 0.879; TLI = 0.898; RMSEA = 0.091; SRMR = 0.142), a need for refinement was detected. We excluded items with low factor loading (*λ* < 0.50; Brazilian sample: exclusion of items 6, 11, and 39; Portuguese sample: exclusion of items 6, 10, 11, 25, and 39). The refined model presented adequate factorial, convergent, and discriminant validity and reliability for both samples (Figure 1). Moderate correlations were observed between Psychological, Health and Physical Condition factors (*r* = 0.55–0.62), both for the Brazilian and Portuguese sample, and between body factor with health and physical condition factors (*r* = 0.58–0.59) only for the Brazilian sample.

Table 4 presents the comparison of the mean scores of each motive-related factor (single-factor models) of the EMI-2 according to sex, exercise level, and weight status. In the Brazilian sample, males had higher scores in the factors “Enjoyment,” “Challenge,” “Social recognition,” “Affiliation” and “Competition” (Psychological and Interpersonal domains) and in the factor “Strength and endurance” (Body domain). Females had the highest scores in the factor “Weight management” of the Body domain and the factors “Health pressures,” “Ill-health avoidance” and “Positive health” of the Health domain. The only factor in which scores were not different between exercisers and non-exercisers was “Ill-health avoidance.” Participants classified as underweight presented lower scores for the factors “Social recognition,” “Competition,” “Weight management,” and “Appearance,” while eutrophic people had higher scores on the “Enjoyment” factor, and overweight/obese people had higher scores on the “Weight management” factor.

**Table 4:** Comparison of the mean scores (±standard deviation) for each factor (single-factor models) of the exercise motivations inventory-2 (EMI-2) according to sex, exercise, and weight status in the Brazilian and Portuguese samples.

	Factors													
	Stress management	Revitalization	Enjoyment	Challenge	Social recognition	Affiliation	Competition	Health pressures	Ill health avoidance	Positive health	Weight management	Appearance	Strength and endurance	Nimbleness
**Brazilian sample**															*Sex*														
Male (*n* = 326)	2.87 ± 1.40	3.30 ± 1.38	3.08 ± 1.55	2.31 ± 1.51	0.93 ± 1.14	1.87 ± 1.44	1.56 ± 1.54	1.64 ± 1.39	3.34 ± 1.46	3.78 ± 1.33	2.64 ± 1.60	2.73 ± 1.42	3.30 ± 1.46	2.57 ± 1.63
Female (*n* = 674)	2.83 ± 1.45	3.20 ± 1.46	2.64 ± 1.61	1.91 ± 1.38	0.54 ± 0.90	1.47 ± 1.38	0.77 ± 1.13	1.98 ± 1.49	3.56 ± 1.33	4.00 ± 1.20	3.20 ± 1.67	2.86 ± 1.34	3.09 ± 1.41	2.65 ± 1.56
Test statistic^†^	*F* = 0.15	*F*_W_ = 0.97	*F* = 17.06	*F* = 17.37	*F*_W_ = 29.60	*F* = 18.72	*F*_W_ = 68.09	*F*_W_ = 12.33	*F*_W_ = 5.58	*F*_W_ = 6.47	*F* = 25.55	*F* = 1.91	*F* = 4.93	*F* = 0.56
*p*	0.698	0.325	<0.001*	<0.001*	<0.001*	<0.001*	<0.001*	0.001*	0.018*	0.011*	<0.001*	0.167	0.027*	0.455
Effect size^‡^	<0.001	0.001	0.017	0.017	0.034	0.018	0.078	0.012	0.006	0.007	0.025	0.002	0.005	0.001
*Exercise*														
Non-exercisers (*n* = 506)	2.54 ± 1.46	2.78 ± 1.48	2.13 ± 1.53	1.68 ± 1.32	0.55 ± 0.92	1.34 ± 1.28	0.76 ± 1.14	2.13 ± 1.52	3.46 ± 1.39	3.74 ± 1.35	2.87 ± 1.70	2.64 ± 1.40	2.83 ± 1.47	2.42 ± 1.62
Exercisers (*n* = 490)	3.14 ± 1.34	3.70 ± 1.21	3.45 ± 1.40	2.41 ± 1.46	0.77 ± 1.06	1.86 ± 1.48	1.30 ± 1.46	1.59 ± 1.36	3.53 ± 1.36	4.12 ± 1.10	3.15 ± 1.62	2.99 ± 1.32	3.50 ± 1.32	2.83 ± 1.53
Test statistic^†^	*F*_W_ = 45.08	*F*_W_ = 113.92	*F*_W_ = 203.08	*F* = 68.33	*F*_W_ = 12.70	*F*_W_ = 34.51	*F*_W_ = 42.05	*F*_W_ = 35.37	*F* = 0.69	*F*_W_ = 24.12	*F* = 6.89	*F*_W_ = 16.78	*F*_W_ = 56.94	*F* = 16.26
*p*	<0.001*	<0.001*	<0.001*	<0.001*	<0.001*	<0.001*	<0.001*	<0.001*	0.405	<0.001*	0.009*	<0.001*	<0.001*	<0.001*
Effect size^‡^	0.043	0.102	0.169	0.064	0.013	0.034	0.041	0.034	0.001	0.024	0.007	0.017	0.054	0.016
*Weight status*														
Underweight (*n* = 94)	2.70 ± 1.42	3.13 ± 1.36	2.48 ± 1.66^a^	1.68 ± 1.23^a^	0.30 ± 0.66^a^	1.48 ± 1.32	0.57 ± 0.90^a^	1.79 ± 1.40	3.22 ± 1.43	3.81 ± 1.37	1.14 ± 1.13^a^	2.24 ± 1.34^a^	2.89 ± 1.36	2.34 ± 1.55^ab^
Eutrophic (*n* = 628)	2.91 ± 1.44	3.31 ± 1.43	2.91 ± 1.60^b^	2.15 ± 1.47^b^	0.69 ± 1.01^b^	1.65 ± 1.44	1.06 ± 1.37^b^	1.82 ± 1.48	3.50 ± 1.39	4.00 ± 1.23	2.98 ± 1.63^b^	2.89 ± 1.38^b^	3.24 ± 1.43	2.73 ± 1.59^b^
Overweight/obese (*n* = 262)	2.70 ± 1.42	3.10 ± 1.47	2.62 ± 1.57^a^	1.96 ± 1.40^ab^	0.75 ± 1.08^b^	1.53 ± 1.37	1.14 ± 1.35^b^	1.97 ± 1.43	3.56 ± 1.30	3.80 ± 1.23	3.79 ± 1.28^c^	2.88 ± 1.30^b^	3.08 ± 1.44	2.45 ± 1.54^a^
Test statistic^†^	*F* = 2.37	*F* = 2.21	*F* = 5.17	*F*_W_ = 6.14	*H* = 15.40	*F* = 1.06	*F*_W_ = 12.80	*F* = 1.10	*F* = 2.17	*F* = 2.64	*F*_W_ = 176.95	*F* = 9.58	*F* = 3.08	*F* = 4.48
*p*	0.094	0.110	0.006*	0.002*	<0.001*	0.348	<0.001*	0.333	0.114	0.075	<0.001*	<0.001*	0.047*	0.012*
Effect size^‡^	0.005	0.004	0.011	0.010	0.016	0.002	0.014	0.002	0.004	0.005	0.181	0.019	0.006	0.009
**Portuguese sample**															*Sex*														
Male (*n* = 100)	2.92 ± 1.32	3.73 ± 1.09	3.72 ± 1.26	2.97 ± 1.28	1.48 ± 1.18	2.13 ± 1.34	2.45 ± 1.36	1.60 ± 1.33	3.14 ± 1.41	4.24 ± 0.82	2.63 ± 1.41	2.98 ± 1.27	3.90 ± 1.02	2.92 ± 1.38
Female (*n* = 219)	3.18 ± 1.16	3.64 ± 1.11	3.32 ± 1.38	2.70 ± 1.24	0.84 ± 0.92	1.67 ± 1.26	1.43 ± 1.26	1.60 ± 1.25	3.28 ± 1.23	4.19 ± 0.87	3.20 ± 1.41	2.86 ± 1.11	3.64 ± 1.07	3.12 ± 1.22
Test statistic^†^	*F* = 3.04	*F* = 0.42	*F* = 5.94	*F* = 3.24	*F*_W_ = 23.64	*F* = 8.88	*F* = 43.25	*F* = 0.00	*F* = 0.75	*F* = 0.26	*F* = 11.40	*F* = 0.74	*F* = 4.02	*F* = 1.67
*p*	0.082	0.513	0.015*	0.073	<0.001*	0.003*	<0.001*	0.997	0.386	0.610	0.001*	0.392	0.046*	0.197
Effect size^‡^	0.009	0.001	0.018	0.010	0.082	0.027	0.120	<0.001	0.002	0.001	0.035	0.002	0.013	0.005
*Exercise*														
Non-exercisers (*n* = 97)	2.70 ± 1.21	3.00 ± 1.19	2.55 ± 1.40	2.30 ± 1.25	0.89 ± 0.94	1.53 ± 1.22	1.45 ± 1.28	1.62 ± 1.24	3.17 ± 1.20	3.87 ± 0.93	2.99 ± 1.42	2.60 ± 1.16	3.27 ± 1.14	2.92 ± 1.38
Exercisers (*n* = 220)	3.27 ± 1.18	3.95 ± 0.93	3.84 ± 1.13	2.98 ± 1.20	1.09 ± 1.08	1.92 ± 1.32	1.86 ± 1.39	1.59 ± 1.29	3.26 ± 1.33	4.35 ± 0.78	3.03 ± 1.45	3.02 ± 1.14	3.92 ± 0.96	3.11 ± 1.23
Test statistic^†^	*F* = 15.29	*F*_W_ = 48.19	*F*_W_ = 63.53	*F* = 21.37	*F*_W_ = 2.80	*F* = 6.32	*F* = 6.04	*F* = 0.06	*F* = 0.32	*F* = 22.48	*F* = 0.05	*F* = 8.74	*F* = 26.79	*F* = 1.51
*p*	<0.001*	<0.001*	<0.001*	<0.001*	0.095	0.012*	0.015*	0.802	0.571	<0.001*	0.817	0.003*	<0.001*	0.220
Effect size^‡^	0.046	0.156	0.192	0.064	0.008	0.020	0.019	<0.001	0.001	0.067	<0.001	0.027	0.078	0.005
*Weight status*														
Underweight (*n* = 27)	3.17 ± 1.02	3.52 ± 1.16	3.02 ± 1.49	2.60 ± 1.18	0.63 ± 0.95ª	1.34 ± 1.12	1.32 ± 1.24	1.41 ± 1.22	2.98 ± 1.34	4.04 ± 0.96	2.13 ± 1.55^a^	2.67 ± 1.32	3.58 ± 1.04	2.89 ± 1.34
Eutrophic (*n* = 227)	3.10 ± 1.24	3.70 ± 1.11	3.57 ± 1.29	2.84 ± 1.26	1.05 ± 1.05^ab^	1.87 ± 1.30	1.75 ± 1.38	1.61 ± 1.29	3.26 ± 1.28	4.26 ± 0.79	3.01 ± 1.43^b^	2.92 ± 1.09	3.78 ± 1.01	3.10 ± 1.25
Overweight/obese (*n* = 53)	3.07 ± 1.25	3.70 ± 1.08	3.32 ± 1.36	2.74 ± 1.30	1.20 ± 1.07^b^	1.81 ± 1.38	2.08 ± 1.45	1.70 ± 1.25	3.27 ± 1.29	4.08 ± 1.06	3.54 ± 1.22^b^	3.02 ± 1.38	3.69 ± 1.23	2.99 ± 1.39
Test statistic^†^	H = 0.03	H = 0.70	*F* = 2.57	*H* = 1.36	*H* = 6.58	*F* = 1.99	*F* = 2.81	*F* = 0.46	*F* = 0.62	*H* = 1.62	*H* = 14.34	*F*_W_ = 0.62	*H* = 1.16	*H* = 0.51
*p*	0.984	0.707	0.079	0.507	0.037*	0.139	0.062	0.630	0.537	0.446	<0.001*	0.540	0.560	0.775
Effect size^‡^	<0.001	0.002	0.017	0.004	0.021	0.013	0.018	0.003	0.004	0.005	0.047	0.005	0.004	0.002

^†^*F*: ANOVA, *F*w: Welchs’s ANOVA, *H*: Kruskal–Wallis; ^ab^Different letters indicate significant statistical difference among groups according to weight status (Dunn *post hoc* test, *α* = 5%); *p < 0.05; ^‡^For ANOVA, the partial eta-squared statistic was computed, for Kruskal–Wallis, epsilon-squared was calculated.

The Portuguese sample showed similar results to the Brazilian sample for men and women. Portuguese exercisers had higher scores on the factors “Stress management,” “Revitalization,” “Enjoyment,” “Challenge,” “Affiliation,” “Competition,” “Positive health,” “Appearance” and “Strength and endurance.” According to weight status, underweight Portuguese students had the lowest score for the “Weight management” factor.

Table 5 shows the results of the comparison between groups for each sample when using the model proposed in this study, considering the five domains and the variables sex, exercise practice, and weight status. In the Brazilian sample, males had higher scores in the Interpersonal domain, while females had higher scores in the Health and Body domains. Exercisers had higher scores in all domains, except for the Health domain, without difference between the groups. Moreover, overweight / obese individuals had the highest score in the Body domain, while underweight individuals had the lowest score in the Interpersonal and Body domain. In the Portuguese sample, the highest scores for men were for the Interpersonal domain, and for exercisers, the highest scores were in the Psychological, Interpersonal, and Physical Fitness domains. Regarding the different weight statuses, individuals with low weight had lower scores in the Body domain than individuals with overweight/obese.

**Table 5:** Comparison of the mean scores (±standard deviation) of each domain (as proposed in this study) of the exercise motivations inventory-2 (EMI-2) according to sex, exercise, and weight status in the Brazilian and Portuguese samples.

	Proposed domains				
	Psychological	Interpersonal	Health	Body	Physical condition
**Brazilian sample**					
*Sex*					
Male (*n* = 326)	3.14 ± 1.36	1.33 ± 1.14	3.01 ± 1.44	2.85 ± 1.44	2.88 ± 1.46
Female (*n* = 674)	2.97 ± 1.42	0.78 ± 0.87	3.29 ± 1.34	3.26 ± 1.49	2.79 ± 1.38
Test statistic^†^	*F* = 3.14	*F*_W_ = 59.22	*F*_W_ = 8.38	*F* = 17.04	*F* = 0.91
*p*	0.076	<0.001*	0.004*	<0.001*	0.340
Effect size^‡^	0.003	0.067	0.009	0.017	0.001
*Exercise*					
Non-exercisers (*n* = 506)	2.53 ± 1.40	0.77 ± 0.89	3.24 ± 1.40	2.96 ± 1.53	2.54 ± 1.43
Exercisers (*n* = 490)	3.53 ± 1.22	1.14 ± 1.07	3.15 ± 1.36	3.29 ± 1.42	3.10 ± 1.32
Test statistic^†^	*F*_W_ = 143.42	*F*_W_ = 36.36	*F* = 1.07	*F* = 12.55	*F*_W_ = 41.97
*p*	<0.001*	<0.001*	0.301	<0.001*	<0.001*
Effect size^‡^	0.126	0.035	0.001	0.012	0.040
*Weight status*					
Underweight (*n* = 94)	2.82 ± 1.40^ab^	0.63 ± 0.70^a^	2.97 ± 1.39	1.75 ± 1.11^a^	2.52 ± 1.35^a^
Eutrophic (*n* = 628)	3.13 ± 1.40^b^	0.98 ± 1.02^b^	3.20 ± 1.40	3.13 ± 1.47^b^	2.92 ± 1.41^b^
Overweight/obese (*n* = 262)	2.87 ± 1.41^a^	1.02 ± 1.03^b^	3.26 ± 1.32	3.63 ± 1.29^c^	2.68 ± 1.38^ab^
Test statistic^†^	*F* = 4.34	*F*_W_ = 10.48	*F* = 1.57	*F*_W_ = 91.44	*F* = 5.10
*p*	0.013*	<0.001*	0.208	<0.001*	0.006*
Effect size^‡^	0.009	0.012	0.003	0.114	0.010
**Portuguese sample**					
*Sex*					
Male (*n* = 100)	3.56 ± 1.09	1.93 ± 1.09	3.40 ± 1.36	3.00 ± 1.20	3.42 ± 1.07
Female (*n* = 219)	3.46 ± 1.11	1.13 ± 0.94	3.33 ± 1.45	3.29 ± 1.25	3.34 ± 1.04
Test statistic^†^	*F* = 0.55	*F* = 45.12	*F* = 0.20	*F* = 3.77	*F* = 0.40
*p*	0.460	<0.001*	0.653	0.053	0.525
Effect size^‡^	0.002	0.125	0.001	0.012	0.001
*Exercise*					
Non-exercisers (*n* = 97)	2.81 ± 1.15	1.16 ± 0.98	3.51 ± 1.36	3.04 ± 1.28	3.03 ± 1.16
Exercisers (*n* = 220)	3.79 ± 0.94	1.47 ± 1.07	3.29 ± 1.45	3.26 ± 1.22	3.51 ± 0.96
Test statistic^†^	*F*_W_ = 54.35	*F* = 6.06	*F* = 1.60	*F* = 2.32	*F*_W_ = 13.16
*p*	<0.001*	0.014*	0.207	0.129	<0.001*
Effect size^‡^	0.167	0.019	0.005	0.007	0.046
*Weight status*					
Underweight (*n* = 27)	3.34 ± 1.15	0.96 ± 0.94^a^	3.65 ± 1.03	2.55 ± 1.45^a^	3.20 ± 1.11
Eutrophic (*n* = 227)	3.55 ± 1.09	1.39 ± 1.06^ab^	3.37 ± 1.44	3.21 ± 1.21^ab^	3.42 ± 1.00
Overweight/obese (*n* = 53)	3.42 ± 1.10	1.61 ± 1.06^b^	3.13 ± 1.44	3.52 ± 1.18^b^	3.36 ± 1.21
Test statistic^†^	*H* = 1.87	*H* = 7.30	*H* = 2.16	*H* = 9.70	*F* = 0.55
*p*	0.392	0.026^*^	0.339	0.008^*^	0.576
Effect size^‡^	0.006	0.024	0.007	0.032	0.004

^†^*F*: ANOVA, *F*w: Welchs’s ANOVA, *H*: Kruskal–Wallis; ^ab^Different letters indicate significant statistical difference among groups according to weight status (Dunn *post hoc* test, *α* = 5%); *p < 0.05; ^‡^For ANOVA, the partial eta-squared statistic was computed, for Kruskal–Wallis, epsilon-squared was calculated.

## Discussion

The psychometric properties of EMI-2 applied to a sample of university students from two Portuguese-speaking countries were verified. A proposal for the use of the instrument was presented considering the theoretical concepts of the original tool, which allowed the evaluation of the motives for the exercise practice, covering its multifactorial aspect and the relationship between factors. This was the first study to test the one-factor model of Klain et al. (2015) on a sample of non-exercisers, widening possibilities of the instrument’ application. Assessing the motives for exercising in non-exercisers may guide strategies for increasing adherence to exercise practice.

As presented by Klain et al. (2015), the single-factor models (Table 2) tested for the Brazilian and Portuguese samples presented adequate fit in all factors. However, single-factor models do not allow the evaluation of motives as a broader concept that reflects different factors. Thus, this work tested a model that considered the structuring of the motive-related concept based on the EMI-2 factors (oblique model; Markland and Ingledew, 1997). An exploratory analysis was performed using the Brazilian sample, which allowed the confirmation of this proposal. The final model had 40 items distributed in five domains. After refinement, the fit of the model was confirmed in the Brazilian and Portuguese samples. In both the Brazilian and Portuguese factor models, a moderate correlation was observed among the Psychological, Health, and Physical Condition factors. Two reasons can be speculated for this result. First, because these factors can be considered intrinsic motives for exercise (Markland and Ingledew, 1997; Dore et al., 2022). Second, because they are associated with better physical (Health and Physical Condition factors) and mental (Psychological and Physical Condition factors) health and well-being (Markland and Ingledew, 1997; Dore et al., 2022). Despite this theoretical approximation of the factors, the correlation was not strong enough to justify a combined evaluation of them. Thus, investigating the factors independently, as presented in the factor model, allows for more targeted and possibly more effective health promotion strategies to be developed.

Another moderate correlation, observed only in the Brazilian sample, was between Body factor and Health and Physical Condition factors. It can be speculated that this is associated with a greater internalization of sociocultural pressures by Brazilians that beauty standards, such as thin women (Frederick et al., 2022b) and thin or athletically built men (Frederick et al., 2022a), are indicative of good health and physical condition. Anic et al. (2022) point out that exercise motives related to body appearance can present potential negative psychological states to the individual, such as body dissatisfaction or low self-esteem. Thus, it becomes relevant that health promotion strategies break sociocultural paradigms related to appearance and health. This requires that they minimize body appearance motives for exercise and emphasize physical and mental health as exercise motives (Anic et al., 2022).

Comparing the fitted factorial models of Brazil and Portugal, no configural invariance was observed between them. Therefore, motive-related factors are operationalized differently in each country. Thus, the confidence of future study results depends on the use of a country- and context-specific model, accounting for the different interpretations of the items and their contribution to motive-related factor for exercise in different cultures and target populations. This issue has been emphasized previously by Caperchione et al. (2011) and Conn et al. (2013) who show that cultural contexts interfere in the individuals’ attitudes toward exercise. For those authors, cultural differences are reflected in the interpretation of the questionnaire and in the perceived benefits of exercise, performed based on specific cultural and social experiences.

Those differences also arise in distinct target populations within the same country. While in our study the first-order factorial model of the EMI-2 with 5 factors and 17 items removed was the one that fitted the sample of Portuguese university students, Rodrigues et al. (2019) observed that the factorial model with 14 first-order factors and 5 s-order factors, with the exclusion of 2 items, fitted to the sample of Portuguese exercisers. It points out that identifying factor models of the EMI-2 that operationalize in certain target populations cannot be extrapolated to others. For researchers, this implies the need to conduct validation studies to identify or confirm factorial models of the EMI-2 that fit to the study sample. Furthermore, if one of the aims is direct comparison between samples from different contexts and/or cultures, it is necessary to ensure the configural and measurement invariance of the factorial models. For health professionals or others who aims to apply the EMI-2 and obtain accurate results in the individual or collective assessment of exercise motives, it is important to define which items and domains/factors of this instrument should be considered. This information can be obtained from validation studies considering the specific target population.

Because of the non-invariance observed between the two countries, the characteristics that influenced the motives for exercise were studied separately in the Brazilian and Portuguese samples. For sex, males of both countries had higher scores in the interpersonal domain. In relation to females, higher scores in the body and health domains were found only in the Brazilian sample. These results are in agreement with the literature (Guedes et al., 2013; Ednie and Stibor, 2017; Rodrigues et al., 2019; Anic et al., 2022), which associates these differences with the social characteristics of each group. While in females a good appearance and a body weight within present beauty standards are seen as positive attributes (Anic et al., 2022), in males, the competitive environment added to the need for social recognition are the motive-related factors for the practice of exercise sociability (Ley, 2020; Valenzuela et al., 2020).

Corroborating the literature (Lores et al., 2004; Guedes et al., 2013), we found that exercisers had higher scores in motive-related factors for exercising in both countries, except for the health factor. These results suggest that the motives for exercise may be related to aspects such as health protection or recovery, weight change, strength development, among others, which are similarly interpreted in the two countries (Gill et al., 2017; Pedersen et al., 2021; Katzmarzyk et al., 2022). In the Portuguese sample, although exercisers had higher scores, the differences between exercisers and non-exercisers were less pronounced than in the Brazilian scenario. Regarding weight status, overweight / obese individuals in both countries had the highest scores in the body domain specifically in the “Weight management” factor. This result may be associated with the medical recommendations for practicing exercises given to these subjects as part of the treatment for the condition (da Silva et al., 2019).

Limitations of the study include the cross-sectional study design, which does not allow to establish cause and effect relationships between the investigated variables, and the convenience sampling strategy. However, the effect of these limitations was minimized by the used of the correct sample size to meet the demand of analytical strategies and the external validity of the data was confirmed using independent Brazilian samples for exploratory and confirmatory analysis. The Portuguese sample size can be considered a limitation because it is not enough to split it into two independent samples and perform exploratory and confirmatory analysis. Thus, future studies larger samples of the Portuguese university students are recommended in order to expand the evidence related to the theoretical model of the EMI-2. Self-reported weight and height measurements for calculating BMI can also be considered a limitation, although self-reported values have shown excellent agreement with measured weight and height and are commonly used in large epidemiological studies (Campos et al., 2018). Moreover, although BMI is the most widely used index to estimate total body fat, it also has some limitations by not assessing specific aspects of body composition (Piqueras et al., 2021). Thus, future studies that include the relationship of the EMI-2 factors with other anthropometric health indicators (Piqueras et al., 2021), such as body fat percentage and fat-free mass index, may be relevant in understanding the exercise motives.

This study contributes to professionals and researchers in the field by providing estimates of the psychometric characteristics of the EMI-2 for use in Portuguese-speaking countries. Our data can also help professionals to better understand the motive-related factors for the practice of exercise, which can be used as a guide for the development of educational and preventive strategies focused on health promotion.

## Conclusion

Although the original proposal of 14 factors (single-factor models) adequately fitted the data of the Brazilian and Portuguese samples, it did not provide a comprehensive assessment of the motives for exercise concept, being limited to each aspect evaluated. Thus, in this study, a five-domain model was tested, showing appropriate fit to the data of samples from both countries and allowing a more comprehensive assessment of the construct. The EMI-2 was not invariant between the two countries indicating specific individualities in the operationalization of this scale.

The motives for exercising were mainly related to psychological and interpersonal factors for men, and to health-related factors for women. Individuals who practiced exercise had higher EMI-2 scores than non-exercisers. For overweight and obese participants, exercise is related to body factors. Therefore, these characteristics should be considered when assessing the motives for exercise for the development of tailored protocols.

## Data availability statement

The original contributions presented in the study are included in the article/Supplementary material, further inquiries can be directed to the corresponding author.

## Ethics statement

The studies involving human participants were reviewed and approved by Brazil: Research Ethics Committee of the Faculty of Pharmaceutical Sciences - UNESP (CAAE: 63553516.4.0000.5426) Portugal: Ethics Committee of the ISPA Research Center – University Institute (D/009/10/2018). The patients/participants provided their written informed consent to participate in this study.

## Author contributions

All authors contributed substantially to the work. AS and JC contributed to the study conception and design. AS and MS contributed to the acquisition of the data. LC, AS, JM, and JC contributed to the analysis and interpretation of the data. LC, AS, and JC contributed to drafting of the article. LC, AS, MS, and JC contributed to revising of the article. LC contributed to editing of the article. All authors approved the final version.

## Funding

This work was supported by grants #2017/04236-0 and 2018/04352-2, São Paulo Research Foundation (FAPESP).

## Conflict of interest

The authors declare that the research was conducted in the absence of any commercial or financial relationships that could be construed as a potential conflict of interest.

## Publisher’s note

All claims expressed in this article are solely those of the authors and do not necessarily represent those of their affiliated organizations, or those of the publisher, the editors and the reviewers. Any product that may be evaluated in this article, or claim that may be made by its manufacturer, is not guaranteed or endorsed by the publisher.
