# Supplementary figures and images for: Exercise motives among university students – A Brazil-Portugal transnational study
Source: Front Psychol. 2022 Nov 14;13:1009762. doi: 10.3389/fpsyg.2022.1009762 (PMC9702053; doi:10.3389/fpsyg.2022.1009762)

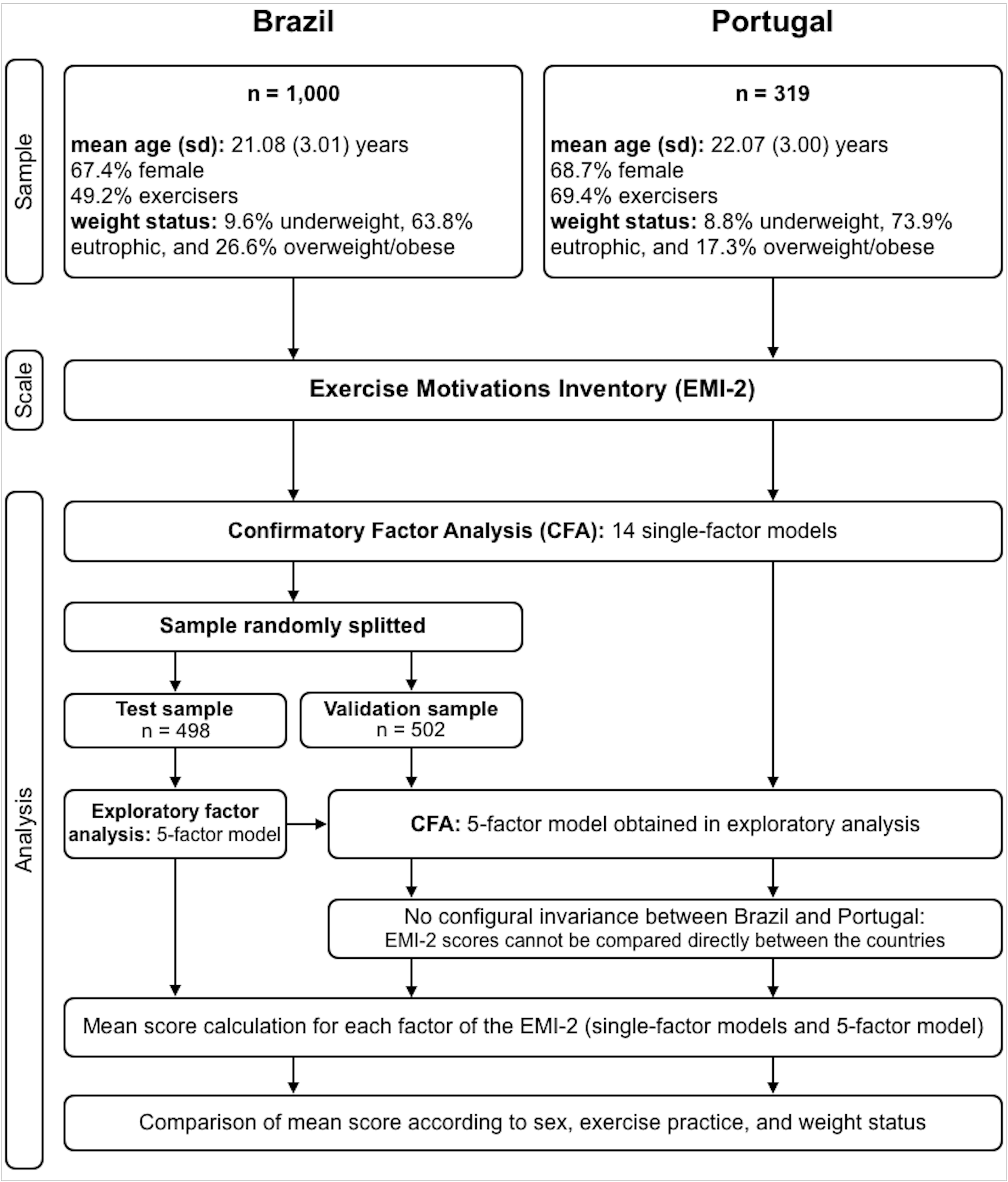

Supplement: Supplementary file 2 [file Image_1.TIFF]
