# Supplementary material for: Exercise motives among university students – A Brazil-Portugal transnational study
Source: Front Psychol. 2022 Nov 14;13:1009762. doi: 10.3389/fpsyg.2022.1009762 (PMC9702053; doi:10.3389/fpsyg.2022.1009762)
Supplement: Supplementary file 3 [file Table_1.DOCX]

Supplemental File 1. Exercise Motivations Inventory-2 (EMI-2).

|  | English Version^†^ | Portuguese Version* |
| --- | --- | --- |
| Instruction | If you think a statement is not at all true to you, cross "0".  If you think a statement is completely true to you, cross "5".  If you think that a statement is partially true to you, cross "1", "2", "3", or "4" according to how strongly that statement reflects the reason why you exercise.  **Personally, I exercise (or may exercise**):  **0 = Not at all true to me;**  **5= Completely true to me** | Se considerar que uma afirmação não é, de maneira nenhuma, verdadeira para si, deverá assinalar "0".  Se entender que a afirmação é completamente verdadeira para si, assinale "5".  Se entender que uma afirmação é parcialmente verdadeira para si, assinale "1", "2", "3" ou "4", de acordo com a intensidade com que essa mesma afirmação reflete o porquê de você praticar exercício físico.  **Pessoalmente, faço exercício físico (ou poderei vir a fazer):**  **0 = Nada verdadeiro para mim;**  **5 = Completamente verdadeiro para mim** |
| Item |  |  |
| 1 | To stay slim | Para me manter elegante |
| 2 | To avoid ill-health | Para evitar ficar doente |
| 3 | Because it makes me feel good | Porque me faz sentir bem |
| 4 | To help me look younger | Para me ajudar a parecer mais novo |
| 5 | To show my worth to others | Para mostrar o meu mérito/valor perante os outros |
| 6 | To give me space to think | Para ter tempo para pensar |
| 7 | To have a healthy body | Para ter um corpo saudável |
| 8 | To build up my strength | Para me fortalecer/tornar mais robusto |
| 9 | Because I enjoy the feeling of exerting myself | Porque gosto da sensação de me exercitar |
| 10 | To spend time with friends | Para passar tempo com os amigos |
| 11 | Because my doctor advised me to exercise | Porque o meu médico me aconselhou |
| 12 | Because I like trying to win in physical activities | Porque gosto de tentar ganhar/vencer quando faço atividade física |
| 13 | To stay / become more agile | Para me manter/tornar mais ágil |
| 14 | To give me goals to work towards | Para ter objetivos que orientem o meu esforço |
| 15 | To lose weight | Para perder peso |
| 16 | To prevent health problems | Para prevenir problemas de saúde |
| 17 | Because I find exercise invigorating | Porque o exercício físico é revigorante |
| 18 | To have a good body | Para ter um corpo bonito |
| 19 | To compare my abilities with other peoples’ | Para comparar as minhas capacidades com as dos outros |
| 20 | Because it helps reduce tension | Porque ajuda a reduzir a tensão |
| 21 | Because I want to maintain good health | Porque pretendo manter uma boa saúde |
| 22 | To increase my endurance | Para aumentar/melhorar a minha resistência |
| 23 | Because I find exercising satisfying in and of itself | Porque o exercício físico me satisfaz tanto no momento da prática como fora dela |
| 24 | To enjoy the social aspects of exercising | Para tirar partido/desfrutar dos aspectos sociais inerentes à prática do exercício físico |
| 25 | To help prevent an illness that runs in my family | Para ajudar a prevenir uma doença familiar/hereditária |
| 26 | Because I enjoy competing | Porque gosto de competir |
| 27 | To maintain flexibility | Para manter a flexibilidade |
| 28 | To give me personal challenges to face | Porque me proporciona situações desafiantes |
| 29 | To help control my weight | Para me ajudar a controlar o peso |
| 30 | To prevent heart disease | Para evitar doenças do coração |
| 31 | To recharge my batteries | Para recarregar energias |
| 32 | To improve my appearance | Para melhorar a minha aparência |
| 33 | To gain recognition for my accomplishments | Para ser reconhecido pelas minhas prestações/realizações |
| 34 | To help manage stress | Para ajudar a gerir/controlar o stress |
| 35 | To feel more health | Para me sentir mais saudável |
| 36 | To get stronger | Para me tornar mais forte |
| 37 | For enjoyment of the experience of exercising | Por gostar da experiência de praticar exercício físico |
| 38 | To have fun being active with other people | Para me divertir estando ativo com outras pessoas |
| 39 | To help me recover from an illness / injury | Para me ajudar a recuperar de uma doença/lesão |
| 40 | Because I enjoy physical competition | Porque gosto da competição física |
| 41 | To stay/ become flexible | Para me manter/tornar flexível |
| 42 | To develop personal skills | Para desenvolver habilidades/capacidades pessoais |
| 43 | Because exercise helps me burn calories | Porque o exercício físico ajuda-me a queimar calorias |
| 44 | To look more attractive | Para ter um aspecto mais atrativo |
| 45 | To accomplish things that others are incapable of | Para alcançar coisas que os outros não conseguem |
| 46 | To release tension | Para libertar a tensão |
| 47 | To develop my muscles | Para desenvolver os músculos |
| 48 | Because I feel at my best when I exercising | Porque me sinto na minha melhor forma quando faço exercício físico |
| 49 | To make new friends | Para fazer novos amigos |
| 50 | Because I find physical activities fun, especially competition is involved | Porque acho o exercício físico divertido, especialmente quando envolve competição |
| 51 | To measure myself against personal standards | Para me avaliar em função de critérios pessoais |

^†^ Markland D, Ingledew DK. The measurement of exercise motives: Factorial validity and invariance across gender of a revised Exercise Motivations Inventory. British Journal of Health Psychology. 1997;2(4):361-76.

* Klain IP, Matos DGD, Cid L, Aidar FJ, Leitão JC, Moutão JM. Evidências de validade da versão brasileira do Exercise Motivation Inventory-2 em contexto de academia e personal training. Motricidade. 2015;11(2):62-74.
